# *trans*-Cinnamaldehyde as a Novel Candidate to Overcome Bacterial Resistance: An Overview of In Vitro Studies

**DOI:** 10.3390/antibiotics12020254

**Published:** 2023-01-27

**Authors:** Federica Usai, Antonella Di Sotto

**Affiliations:** Department of Physiology and Pharmacology “V. Erspamer”, Sapienza University of Rome, P.le Aldo Moro 5, 00185 Rome, Italy

**Keywords:** essential oils, superbugs, antibiotic resistance, terpene, synergism, antibacterial, cefotaxime, ciprofloxacin, fractional inhibitory concentration index

## Abstract

The increasing of drug-resistant bacteria and the scanty availability of novel effective antibacterial agents represent alarming problems of the modern society, which stimulated researchers to investigate novel strategies to replace or assist synthetic antibiotics. A great deal of attention has been devoted over the years to essential oils that contain mixtures of volatile compounds and have been traditionally exploited as antimicrobial remedies. Among the essential oil phytochemicals, remarkable antimicrobial and antibiotic-potentiating activities have been highlighted for cinnamaldehyde, an α,β-unsaturated aldehyde, particularly abundant in the essential oils of *Cinnamomum* spp., and widely used as a food additive in industrial products. In line with this evidence, in the present study, an overview of the available literature has been carried out in order to define the bacterial sensitizing profile of cinnamaldehyde. In vitro studies displayed the ability of the substance to resensitize microbial strains to drugs and increase the efficacy of different antibiotics, especially cefotaxime, ciprofloxacin, and gentamicin; however, in vivo, and clinical trials are lacking. Based on the collected findings, cinnamaldehyde appears to be of interest as an adjuvant agent to overcome superbug infections and antibiotic resistance; however, future more in-dept studies and clinical investigations should be encouraged to clarify its efficacy and the mechanisms involved.

## 1. Introduction

The discovery of antibiotics is considered one of the most important achievements in the history of medicine since their use has significantly reduced morbidity and mortality associated with bacterial infections [1]. However, their inappropriate use and abuse have led to the emergence of antibiotic resistance at an alarming rate, which has resulted in drug treatment failure and the development of recurrent infections [2]. This phenomenon has been favored by incorrect prescriptions and a lack of adherence to therapies [3,4]. Approximately 700,000 people die every year due to infections caused by multidrug-resistant bacteria (MDR), and this number is expected to exceed 10 million deaths by 2050 [2]. 

An irresponsible use of antimicrobial agents has also been highlighted in veterinary and agricultural fields. In fact, large volumes of antibiotics, often unnecessary, are administered to food-producing animals, endangering human health due to the possible presence of drug residues in food and the selection of resistant bacteria [3]. 

Resistant bacteria, also known as superbugs, have limited treatment options, thus representing a serious threat to public health, and increasing the risk of death, especially in critically ill patients, immunocompromised subjects, and in the hospital setting [3,5]. The most severe chronic infections are frequently caused by six pathogenic bacteria, known by their acronym ESKAPE, which means *Enterococcus faecium*, *Staphylococcus aureus*, *Klebsiella pneumoniae*, *Acinetobacter baumannii*, *Pseudomonas aeruginosa* and *Enterobacteria* sp. [6]. Indeed, hospital infections caused by *P. aeruginosa* and *A. baumanii* are resistant to almost all antibiotics; additionally, the extended-spectrum of β-lactamases of the Enterobacteriaceae has limited the efficacy of the latest generations of penicillin and cephalosporins [7]. The loss of drug efficacy along with the emergence of novel superbugs increased the need for innovative therapies; particularly, substances able to increase the susceptibility of bacteria to drugs, thus acting as bacterial sensitizers, have been approached as promising strategies to overcome antibiotic resistance and achieve the expected antibacterial efficacy [8,9]. 

Many natural products, both phytocomplexes and pure compounds, have been studied as possible antibacterial and sensitizing agents [10]. Among them, essential oils, which are mixtures of naturally occurring volatile compounds with a characteristic smell and flavor, attracted a great deal of attention [11]. Terpenes represent the most abundant compounds of essential oils, with lower amounts of aromatic and aliphatic substances (e.g., aldehydes, phenols, alcohols, and heterocycles) [12]. 

Essential oils are known to possess a broad spectrum of bioactivities, including antimicrobial, anti-inflammatory, antioxidant, genoprotective, and antiproliferative [13,14,15,16,17,18,19,20,21]; The antimicrobial properties of essential oils have been known since antiquity and represent the most exploited up until now. They may act as both bacteriostatic and bactericide agents, being able to inhibit bacterial growth, thus blocking the bacteria’s reproductive ability, and to kill bacterial cells [22,23,24]. Usually, these effects are explained based on the lipophilicity of the essential oil constituents, especially monoterpenes, which can cross the bacterial wall and alter the cell permeability [11,24]. Moreover, they can alter the conformation of different fatty acids, polysaccharides, and phospholipid layers, causing disintegration of the bacterial cell wall [11,24]. These events can be reflected in membrane potential changes, disruption of transporters, and intracellular content leakage, which eventually lead to cell lysis and death [11]. The complex composition of essential oils also allows for hypothesizing the involvement of additional antimicrobial mechanisms, including the inhibition of bacterial enzymes and the interference with systems involved in energy production and the synthesis of structural components [24].

**Figure 1:**
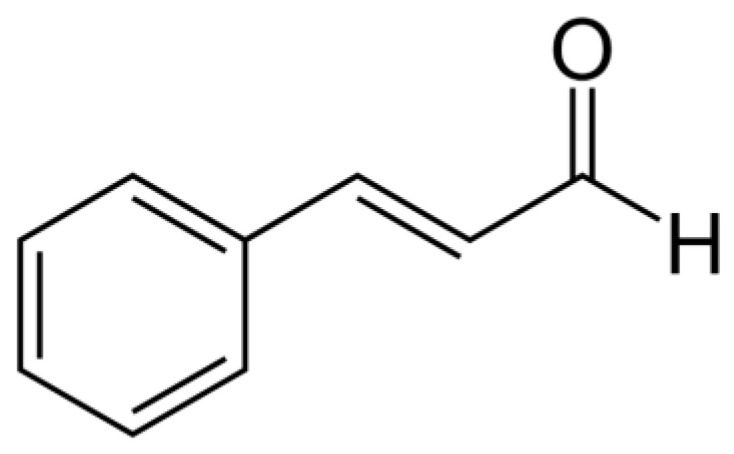
Chemical structure of cinnamaldehyde.

Among the essential oil compounds, a great interest has been devoted to cinnamaldehyde, also known as cinnamic aldehyde or 3-phenyl-2-propenal (Figure 1), an α,β-unsaturated aldehyde, belonging to the class of phenylpropanoids. It is widely used as a food additive in industrial products, such as drinks, candies, ice cream, chewing gum, and condiments [25], and it is rated safe (GRAS) by the United States Food and Drug Administration (FDA) and by the Flavor and Extract Manufacturer’s Association (FEMA) [5]. The FDA and the Council of Europe have recommended a daily intake of 1.25 mg/kg [25]. 

Cinnamaldehyde occurs naturally as a *trans* stereoisomer, namely (2*E*)-3-phenylprop-2-enal or *trans*-cinnamaldehyde, which is especially abundant in the essential oils from *Cinnamomum* spp. (Fam. Lauraceae), where it contributes to the typical aroma [26,27]. However, minor amounts (≤0.9%) of (2*Z*)-cinnamaldehyde in the essential oils of *Cinnamomum* spp. from Madagascar have been reported as well [28].

The bark of *Cinnamomum cassia* Nees (or Chinese cinnamon) and *Cinnamomum verum* J. Presl (or true cinnamon), which achieve about 85% and 90% content of *trans*-cinnamaldehyde, are considered its major natural sources [29], although other varieties (Table 1) can produce high amounts of the substance [30,31,32,33,34,35,36,37,38,39,40,41,42,43,44,45,46,47,48,49]. 

The biosynthesis of cinnamaldehyde arises from the deamination of l-phenylalanine into a cinnamic acid by a phenylalanine-ammonia lyase, followed by the conversion into a cinnamoyl-CoA, mediated by a 4-coumarate-CoA ligase, which in turn is reduced to cinnamaldehyde by a cinnamoyl-CoA reductase [50].

**Figure 2:**
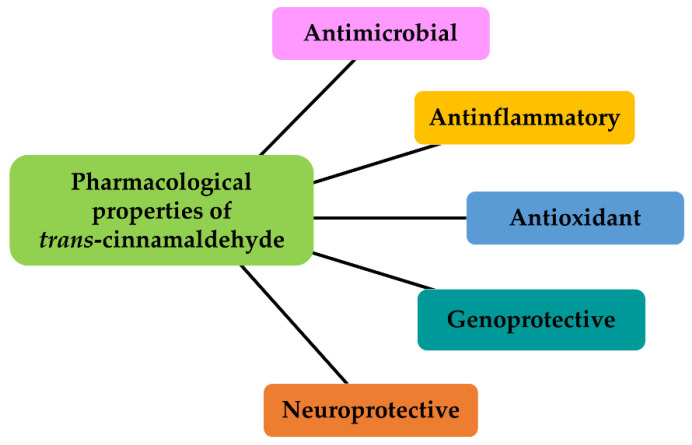
Pharmacological properties of cinnamaldehyde.

Cinnamoyl moiety is a characteristic scaffold of cinnamaldehyde and its derivatives: it is considered as a Michael acceptor due to the presence of a α,α-unsaturated carbonyl pharmacophore, which can react with different electrophilic structures (e.g., enzymes, receptors), leading to several pharmacological effects [28]. Indeed, the substance has been found endowed with remarkable bioactivities in preclinical models (Figure 2), including antioxidant, anti-inflammatory, antimutagenic, antiproliferative, and neuroprotective ones [51,52]; moreover, its chemopreventive power has been reported [53]. Semisynthetic derivatives (e.g., α-hexylcinnamaldehyde) of cinnamaldehyde have also been studied to exploit the pharmacological properties of the lead compound and achieve improvements in its chemical stability [54,55,56]. 

Among the bioactivities of cinnamaldehyde, a remarkable broad spectrum of antibacterial and antifungal properties has been highlighted: the substance was especially effective against *Escherichia coli*, *Staphylococcus aureus*, *Salmonella* spp., and *Bacillus* spp. strains, acting through bactericidal mechanisms [28]. 

The antimicrobial capacity seems to arise from the ability of the substance to interact with the bacterial wall and disrupt its integrity; indeed, the aldehydic group can be easily absorbed by the hydrophilic group of the bacterial surfaces, then it can pass through the cell wall and start a process of inhibition and sterilization by destroying the polysaccharide structure, leading to leakage of ions, proteins, and nucleic acids [25,28]. Other mechanisms, such as the inhibition of biofilm formation and ATP production, along with the interference with the quorum sensing systems, have been reported too [28]. A special attention has also been devoted in the years to the antibiotic-potentiating properties of cinnamaldehyde, especially in superbug strains. In line with this evidence, in the present study, an overview of the available literature has been carried out in order to define the bacterial sensitizing profile of cinnamaldehyde and to highlight a future interest in this natural substance as a novel strategy to overcome antibiotic resistance.

## 2. Methodology

The existing literature in PubMed and Scopus databases was searched in November 2022 to select journal articles over a 20-year period (2002-today) focused on the antibacterial combination of cinnamaldehyde and antibiotics in resistant bacteria; combinations with antifungal agents have also been considered. English was chosen as the preferred language. The keywords “*trans*-cinnamaldehyde”, “cinnamaldehyde” “antibiotic”, “synergism”, and “combination”, and their combinations through the Boolean logical operator “AND” have been used. As a research strategy, the PRISMA methodology was applied to select eligible papers for the study [57]. Notably, the studies focused on herbal extracts or essential oils containing cinnamaldehyde, but not on the pure compound, along with studies in which the purity of the substance was low (<90%) or not specified, and studies assessing other substances, diverse bioactivities, or lacking data, were excluded.

## 3. Results

**Figure 3:**
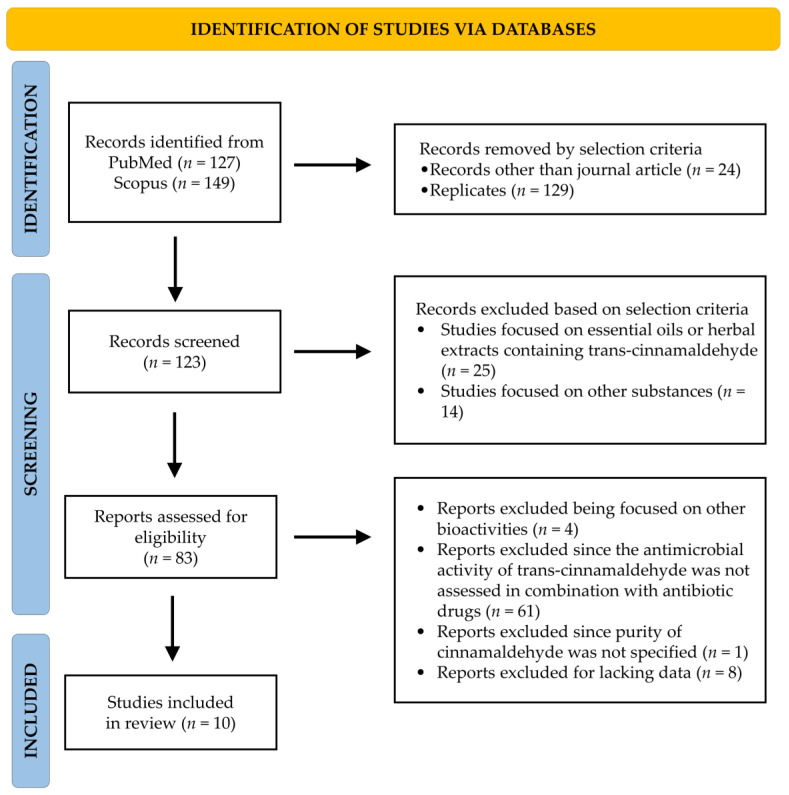
Study selection by PRISMA flow diagram about the ability of cinnamaldehyde to synergize antimicrobial drugs against superbugs.

A total of 276 studies focused on the ability of cinnamaldehyde to potentiate the effect of antimicrobial drugs when used in combination (Figure 3). Among them, 24 records were removed as publications other than journal articles, while 129 were replicates in searched databases. 

The screened 123 papers were further selected; out of which 25 studies focused on essential oils or herbal extracts containing cinnamaldehyde, and the other 14 on other substances, and were excluded. Moreover, out of 83 eligible papers, 4 reports were not included since they focused on other bioactivities; similarly, 61 records evaluating the antimicrobial properties of cinnamaldehyde alone, but not in combination with antimicrobial drugs, were removed too. Furthermore, 8 studies were not included for lacking data and another one since purity was not specified. At the end of the literature analysis, a total of 10 studies were considered eligible since they met the inclusion criteria. 

Based on the selected studies, cinnamaldehyde has been found to be able to potentiate the antimicrobial properties of different drugs, although with specific potency and efficacy with respect to the drug and bacterial (or fungal) strain [58,59,60,61,62,63,64,65,66,67]. Usually, it produces synergistic or additive effects and allows for a significant reduction in the MIC (minimal inhibitory concentration) value of the combined drug, thus suggesting promising bacterial sensitizing properties. It is noteworthy that some of the susceptible bacteria [60,62,63,64,65] belonged to the ESKAPE group (*Enterococcus faecium*, *Staphylococcus aureus*, *Klebsiella pneumoniae*, *Acinetobacter baumannii*, *Pseudomonas aeruginosa*, *Enterobacter* sp.), known to be responsible for resistant infections.

To quantify the type of interaction (synergism or antagonism) between cinnamaldehyde and antibiotic drugs, the fractional inhibitory concentration index (FICI), which represents the sum of FIC concentrations of each component in the mixture, has been conserved [68]. Considering a given combination of two drugs A and B, FIC_A_ is the MIC of drug A in the presence of compound B divided by the MIC of drug A alone (FIC_A_ = [MIC_A_(B)/MIC_A_]), and vice versa for FIC_B_ (FIC_B_ = [MIC_B_(A)/MIC_B_]) [68]. The FICI value is the sum of FIC_A_ and FIC_B_ and reveals the degree of drug interaction: a lower than 0.5 FICI value indicates a synergistic interaction, values between 0.5 and 1 denote additive effects, while FICI values higher than 1 and 4 correspond to null and antagonistic effects, respectively [68].

The ability of cinnamaldehyde to affect drug efficacy in different bacterial strains (Gram-positive and Gram-negative) and fungi has been described and detailed in Table 2. For each microorganism, the strain, antibiotic drug, MIC value (expressed as µg/mL) of the antibiotic drug alone and in combination with cinnamaldehyde, the cinnamaldehyde concentration in combination (expressed as µg/mL), and the FICI value have been displayed. 

### 3.1. Potentiating Effects of Cinnamaldehyde in Gram-Positive Bacteria

The potentiating effects of cinnamaldehyde were evaluated in different Gram-positive bacteria, including *Listeria monocytogenes*, *Staphylococcus aureus* and its methicillin-resistant strains, namely MRSA (methicillin-resistant *S. aureus*), and *Streptococcus pyogenes* (Table 2).

*L. monocytogenes* is a ubiquitous bacterium, implicated within the past decade in several outbreaks of foodborne disease [69]. It causes invasive syndromes, and case fatalities can be around 30% in specific high-risk population groups, such as the elderly, immunocompromised individuals, fetuses, and newborns [70]. Moreover, it may acquire antibiotic resistance genes from the plasmids and conjugative transposons of other organisms [71]. Only a few studies have evaluated the ability of cinnamaldehyde to synergize antibiotics in *L. monocytogenes*. Alves et al. [58] highlighted that the substance produced synergistic effects with nisin (0.50 FICI), a bacteriocin produced by *Lactococcus lactis* strains, reducing the MIC value by 4 folds. 

*S. aureus* is a Gram-positive opportunistic pathogen that is responsible for many nosocomial and community-acquired infections. The attachment to medical implants and host tissue, and the establishment of a mature biofilm, all play an important role in the persistence of chronic infections [72,73]. Clinical use of methicillin led to the development of methicillin-resistant *S. aureus* (MRSA) strains [74], which increased the need for new therapeutic strategies to sensitize these strains to the antibiotic treatment.

Cinnamaldehyde has been assessed against *S. aureus* in association with conventional antibiotics and other antibacterial substances, such as nisin. In particular, two studies have highlighted the ability of cinnamaldehyde to significantly synergize nisin with 0.26 to 0.50 FICI values [58,59]. Remarkable synergistic effects were reported in combination with ampicillin, piperacillin, and bacitracin (0.24–0.37 FICI), with antibiotic MIC values reduced by about 8 folds [60]. The substance was also found to greatly synergize amikacin, amoxicillin, and gentamicin (0.19–0.50 FICI) in MRSA strains [61]; moreover, it lowered by about 2-fold the MIC value of ampicillin and ceftazidime (1.00 FICI), although without exhibiting synergistic effects [61]. Both additive and synergistic interactions were recorded in combination with cefoxitin, oxacillin, and vancomycin [61].

**Table 2:** Effect of cinnamaldehyde in combination with antimicrobial drugs in bacterial strains.

Bacteria	Strain	Antibiotic	MIC [µg/mL]	Antibiotic and *trans*- Cinnamaldehyde Combination		FICI/Type of Interaction	Ref.
				Cinnamaldehyde Concentration [µg/mL]	MIC [µg/mL] (RR)		
Gram-positive							
*Listeria monocytogenes*	ATCC 15313	Nisin	125	16.25	62.5 (4)	0.50/Synergism	[58]
*Staphylococcus aureus*	JL10001	Nisin	16	50	2 (8)	0.32/Synergism	[59]
	JL10002, JL10006, JL10008, JL10011		16	62.5	1 (16)	0.31/Synergism	
	JL1000, JL10005, JL10009, JL10013		32	125	2 (16)	0.31/Synergism	
	JL10004		16	125	2 (8)	0.37/Synergism	
	JL10007 JL10012		16	62.5	2 (8)	0.37/Synergism	
	JL10010		32	62.5	4 (8)	0.37/Synergism	
	ATCC 29213		32	50	2 (16)	0.26/Synergism	
	ATCC 25923		110	25	27.5 (4)	0.50/Synergism	[58]
	bla Z	Ampicillin	32	41.3	4 (8)	0.25/Synergism	[60]
		Bacitracin	32	41.3	4 (8)	0.24/Synergism	
		Piperacillin	128			0.37/Synergism	
Methicillin-resistant *Staphylococcus aureus* (MRSA)	ATCC 33571	Amikacin	31.2	31.25	7.8 (4)	0.38/Synergism	[61]
	Dps-1		31.2	31.25	3.9 (8)	0.25/Synergism	
	Dps-3		62.5	31.25	3.9 (16)	0.19/Synergism	
	ATCC 33571	Amoxicillin	62.5	125	7.8 (8)	0.63/Additive effect	
	Dps-1		125	62.5	31.25 (4)	0.5/Synergism	
	Dps-3		125	31.25	15.6 (8)	0.25/Synergism	
	ATCC 33571	Ampicillin	62.5	125	31.25 (2)	1.00/Additive effect	
	Dps-1		31.3	125	7.8 (4)	0.75/Additive effect	
	Dps-3		62.5	125	15.6 (4)	0.75/Additive effect	
	ATCC 33571	Cefoxitin	31.2	125	7.8 (4)	0.75/Additive effect	
	Dps-1		62.5	125	7.8 (8)	0.62/Additive effect	
	Dps-3		250	31.25	31.25 (4)	0.50/Synergism	
	ATCC 33571	Ceftazidime	125	125	62.5 (2)	1.00/Additive effect	
	Dps-1		125	125	62.5 (2)	1.00/Additive effect	
	Dps-3		250	125	62.5 (4)	0.75/Additive effect	
	ATCC 33571	Gentamicin	3.9	125	0.97 (4)	0.75/Additive effect	
	Dps-1		125	31.25	31.25 (4)	0.37/Synergism	
	Dps-3		250	62.5	62.5 (4)	0.50/Synergism	
	ATCC 33571	Oxacillin	62.5	125	15.6 (4)	0.75/Additive effect	
	Dps-1		500	125	250 (2)	1.00/Additive effect	
	Dps-3		500	31.25	62.5 (8)	0.25/Synergism	
	ATCC 33571	Vancomycin	250	31.25	31.25 (8)	0.25/Synergism	
	Dps-1		250	125	125 (2)	1.00/Additive effect	
	Dps-3		500	125	250 (2)	1.00/Additive effect	
*Streptococcus pyogenes*	erm B	Erythromycin	>512	41.6	>256 (8)	1.00/Additive effect	[60]
		Nitrofurantoin				0.13/Synergism	
Gram-negative							
*Escherichia coli*	28 clinically isolated strains	Cefotaxime	512	0.22	1 (512)	0.07–0.30/75% synergism	[62]
		Ciprofloxacin	512	0.11	8 (64)	0.07–0.50/39.6% synergism	
	ATCC 11775	Erythromycin	16	100	4 (4)	0.50/Synergism	[63]
	ATCC 23739		32	-	-	0.30/Synergism	
	8WT		64	100	16 (4)	0.50/Synergism	
	02:0627		16	100	4 (4)	0.50/Synergism	
	ATCC 23739	Tetracycline	32	-	-	0.30/Synergism	
	ATCC 23739	Novobiocin	128	-	-	0.20/Synergism	
	8WT		64	32	32 (2)	1.00/Additive effect	
	02:0627		128	100	32 (4)	0.50/Synergism	
	ATCC 11775	Bacitracin	>512	-	-	>1.00/Lacking effect	
	ATCC 23739		>512	-	-	>1.00/Lacking effect	
	8WT		>512	-	-	>1.00/Lacking effect	
	02:0627		>512	-	-	>1.00/Lacking effect	
	N00 666	Ampicillin	>512			0.37/Synergism	[60]
		Bacitracin	>512	165.2	>64 (8)	0.63/Additive effect	
		Erythromycin	512	41.3	64 (8)	0.24/Synergism	
		Novobiocin	64	41.3	8 (8)	0.24/Synergism	
		Piperacillin	>512	41.3	>64 (8)	0.24/Synergism	
		Tetracycline	128			0.37/Synergism	
*Klebsiella* sp.	33 clinically isolated strains	Cefotaxime	512	0.05	0.5 (1024)	0.10–0.50/42.4% synergism	[62]
		Ciprofloxacin	512	0.03	2 (256)	0.07–0.50/60.6% synergism	
*Pseudomonas aeruginosa*	PAO1	Carbenicillin	128	396.5	64 (2)	0.75/Additive effect	[64]
		Colistin	7.86	396.5	1.96 (4)	0.50/Synergism	
		Erythromycin	256	396.5	128 (2)	0.75/Additive effect	
		Tobramycin	1443.8	396.5	721.9 (2)	0.75/Additive effect	
		Gentamicin	4.0	7.5	0.25 (16)	0.37/Synergism	[65]
*Salmonella typhimurium*	SGI 1	Ampicillin	>512	41.3	>64 (8)	0.25/Synergism	[60]
		Bacitracin	>512	41.3	>64 (8)	0.24/Synergism	
		Erythromycin	1024	41.3	128 (8)	0.24/Synergism	
		Novobiocin	256	41.3	32 (8)	0.24/Synergism	
		Piperacillin	>512	165.2	>64 (8)	0.63/Additive effect	
		Tetracycline	64			0.37/Synergism	
Fungi							
*Aspergillus fumigatus*	MTCC 2550	Fluconazole	200	5	25 (8)	0.19/Synergism	[66]
*Malassezia pachydermatis*	30 isolated strains	Clotrimazole	0.03–64 (GM 4.5)	1.25–40 (GM 3.15)	0.063–8 (GM 0.52)	0.064–2.125 (GM: 0.52)/40% synergism 60% null effect	[67]
		Fluconazole	1–64 (GM 9.4)	1.25–40 (GM 6.64)	0.25–16 GM 0.7 (4)	0.066–12 (GM 0.73)/ 26.6% synergism 70% antagonism	
		Ketoconazole	0.015–4 (GM 0.08)	1.25–160 (GM 5.48)	0.016–0.062 (GM 0.02)	0.093–6.006 (GM 1.55)/ 23.3% synergism 30% null effect 46,6% antagonism	
		Itraconazole	0.0039–1 (GM 0.02)	1.25–160 (GM 4.66)	0.016–0.125 (GM 0.02)	0.007–16.52 (GM: 0.85)/ 30.0% synergism 56.6% null effect 13.3% antagonism	
		Miconazole	0.03–64 (GM 8.96)	1.25–40 (GM 2.17)	0.016–8 (GM 0.72)	0.039–2.003 (GM: 0.31)/ 66.6% synergism 33.3% null	
		Nystatin	4–64 (GM 41.96)	1.25–20 (GM 2.22)	0.25–64 (GM 29.2)	0.062–1.25 (GM 0.31)/ 70% synergism 30% null effect	
		Terbinafine	0.03–64 (GM 2.57)	1.25–40 (GM 8.31)	0.125–8 (GM 0.29)	0.046–4.5 (GM: 0.97)/ 16.6% synergism 70% null effect 13.3% antagonism	
*Trichophyton rubrum*	IO A-9	Fluconazole	200	1.25	25 (8)	0.16/Synergism	[66]

At last, possible potentiating effects of cinnamaldehyde were evaluated in *Streptococcus pyogenes*, which is an exclusive human Gram-positive bacterial pathogen, characterized by high virulence and mortality risk [75]. Palaniappan et al. [60] highlighted synergistic effects of cinnamaldehyde with nitrofurantoin (0.13 FICI value) in *Streptococcus pyogenes,* while an additive effect was observed in combination with ampicillin (1.00 FICI).

### 3.2. Potentiating Effects of Cinnamaldehyde in Gram-Negative Bacteria

The substance was assessed in combination with different antibiotics in many Gram-negative strains, including *Escherichia coli*, *Klebsiella* spp., *Pseudomonas aeruginosa*, and *Salmonella typhimurium* (Table 2). *E. coli* and *Klebsiella* spp., belonging to the Enterobacteriaceae, are usually part of the intestinal flora but can also contribute to a wide range of both community- and hospital-acquired infections [76]. *Klebsiella* spp. are also responsible for opportunistic nosocomial infections, with a high incidence of resistant strains [77,78]. β-Lactam antibiotics are usually administered to treat their infections, although the resistance to these drugs causes serious pharmacological and medical issues [76]. *E. coli* belongs to the resident flora in the lower intestinal tract of warm-blooded animals, such as humans, but can also be found as an environmental contaminant as a consequence of the release of feces or wastewater effluent [77]. 

Dhara et al. [62] showed that cinnamaldehyde synergized ciprofloxacin (0.07–0.50 FICI) and cefotaxime (0.10–0.50 FICI) in *Klebsiella* spp. in 60.6% and 42.4% of cases, respectively; the MIC values of ciprofloxacin and cefotaxime were lowered by 256 and 1024 folds, respectively. Furthermore, cinnamaldehyde exhibited synergistic effects (≤0.5 FICI) in combination with erythromycin, tetracycline, cefotaxime, ciprofloxacin, ampicillin, and piperacillin in *E. coli*, although with weak or null effects in combination with novobiocin and bacitracin [60,63]. 

As for *Pseudomonas aeruginosa*, a common Gram-negative environmental organism that can cause severe infections in humans owing to its natural resistance to antibiotics and the ability to form biofilms [79], Topa et al. [64] demonstrated that cinnamaldehyde produced synergistic effects with colistin (0.50 FICI) and additive effects with carbenicillin, tobramycin, and erythromycin (0.75 FICI). Recently, Chada et al. [65] highlighted a synergist interaction of cinnamaldehyde with gentamicin in *P. aeruginosa* (0.375 FICI), with a 4-fold lowering of the antibiotic MIC. Moreover, the substance exhibited a quorum quenching (QQ) potential, being able to attenuate the quorum sensing (QS) circuits, particularly by downregulating QS genes and abrogating the biosynthesis of key factors involved in bacterial virulence and biofilm formation [65]. The antivirulence properties of cinnamaldehyde in combination with gentamicin were also confirmed in a *Caenorhabditis elegans* model infected with a *P. aeruginosa* infection [65]. These findings highlight an interest in cinnamaldehyde as a possible anti-quorum sensing agent to be exploited in combination with antibiotics in the battle against *P. aeruginosa* and deserve further in vivo studies for confirmation.

At last, the possible synergistic potential of cinnamaldehyde has been evaluated in *S. typhimurium* in combination with different antibiotics [60]. This bacterium primarily affects the intestinal lumen and often causes diarrhea in infants and young children, leading to food poisoning. Furthermore, the development of drug resistance by *S. typhimurium* strains led to serious complications in clinical patients [80]. Palaniappan et al. [60] showed remarkable synergistic effects of cinnamaldehyde in combination with ampicillin, tetracycline, erythromycin, bacitracin, and novobiocin (0.24–0.37 FICI) in *S. typhimurium*, reducing the MIC values of all the tested antibiotics by about 8 folds. 

### 3.3. Potentiating Effects of Cinnamaldehyde in Fungi

Cinnamaldehyde has also been assayed as a possible strategy to counteract fungi infections, and some studies highlighted its ability to potentiate the effects of some antifungal drugs (Table 2): particularly, it partly synergized azole drugs in *Aspergillus fumigatus*, *Trichophyton rubrum,* and *Malassezia pachydermatis* fungi, being especially effective in combination with fluconazole (<0.2 FICI) [66,67].

## 4. Discussion

The increasing prevalence of drug-resistant bacteria and the lack of effective antibiotics have highly alarmed the scientific community, leading researchers to investigate natural substances as novel strategies to both directly affect bacterial infections and synergize synthetic antibiotics. Among natural compounds, cinnamaldehyde attracted special attention owing to its antibacterial properties and the ability to resensitize microbial strains to drugs [60], thus suggesting a possible interest in the battle against antibiotic resistance. 

In this study, we selected ten in vitro studies, which are not available in vivo or in clinical trials, using the following criteria: >90% purity of cinnamaldehyde and combination of this substance with antimicrobial agents to counteract resistant bacteria. The purity of cinnamaldehyde is a key issue, since the presence of impurities in minor compounds can affect the activity of the tested substance, leading to unreliable results.

Based on the selected studies, the most efficient synergism was found when cinnamaldehyde (0.03–0.05 µg/mL) was assessed in combination with cefotaxime or ciprofloxacin in 33 clinical isolates of *Klebsiella* sp; in fact, the MIC values were lowered by 1024 and 256 folds, respectively [62]. Similar results were obtained in 28 clinical isolates of *Escherichia coli*, where cinnamaldehyde (0.11–0.22 µg/mL) lowered the MIC value of cefotaxime by 512 folds, and that of ciprofloxacin by 64 folds [62]. Interesting synergistic effects of cinnamaldehyde were also highlighted in combination with tetracycline in *Escherichia coli*, where a MIC reduction of 4- to 8-fold was registered; similar potentiating effects were produced in combination with erythromycin, novobiocin, ampicillin, and piperacillin [62,63]. 

Cinnamaldehyde also produced synergistic effects in combination with colistin and gentamicin in *Pseudomonas aeruginosa*, reducing the MIC values by 4- and 16-fold, respectively [64,65], and in MRSA strains in combination with amikacin (16-fold reduction of the antibiotic MIC), gentamicin, and vancomycin, followed by oxacillin and amoxicillin; the substance was found effective at concentrations from 31.25 to 62.5 µg/mL, corresponding to 1/8 and 1/4 of the MIC value [61]. Similarly, a notable antibacterial activity of the combination of cinnamaldehyde and nisin (i.e., 25 to 125 µg/mL cinnamaldehyde and 1/8 of the antibiotic MIC) was reported in *S. aureus* [58,59]. The substance (16.25–41.3 µg/mL) also potentiated the antibiotic effects of ampicillin, tetracycline, erythromycin, bacitracin, and novobiocin in *Salmonella typhimurium* and those of nisin (62.5 µg/mL) in *Listeria monocytogenes* ATCC 15313, reducing the MIC value by 4- to 8-fold [58,60]. 

Schlemmer et al. [67] demonstrated a partial synergism between cinnamaldehyde and fluconazole, ketoconazole, itraconazole, clotrimazole, miconazole, terbinafine, and nystatin against *Malassezia pachydermatis*. Additionally, potentiating effects towards fluconazole (25 µg/mL corresponding to 1/8 of MIC) were reported in *Aspergillus fumigatus* and *Trichophyton rubrum* [66]. Additive effects were achieved when cinnamaldehyde (at a halved MIC value) was administered in combination with ampicillin or cefotaxime in *Staphylococcus aureus* [61], piperacillin in *Salmonella typhimurium* [60], erythromycin in *Streptococcus pyogenes* [60], and bacitracin against *Escherichia coli* [60,61]. Null or antagonistic effects of cinnamaldehyde with some antimicrobial agents were reported as well [63,67].

In this respect, Tetard et al. [81] showed that cinnamaldehyde (>256 µg/mL) triggers an upregulation of the efflux pumps of the resistance-nodulation-cell division (RND) family in *P. aeruginosa*, especially of the multidrug efflux system MexAB-OprM, which can lead to increased drug extrusion and lowered antibiotic efficacy. This effect was found to be transient and persistent until the compound is degraded into cinnamic alcohol, which lacks the ability to induce the efflux pumps [81]. Moreover, the authors highlighted that the resistance induced by cinnamaldehyde in *P. aeruginosa* was modest and gained after several days of exposure at concentrations higher than 900 µg/mL [82]. Furthermore, the mutation mechanisms and the clinical impact remain to be clarified. It is important to outline that the concentrations of cinnamaldehyde inducing bacterial sensitization in *P. aeruginosa* [64] were at least 3- to 120-fold lower than those responsible for the resistance, suggesting that opposite effects can occur depending on the concentrations of the substance; more in-depth studies could clarify this issue.

**Figure 4:**
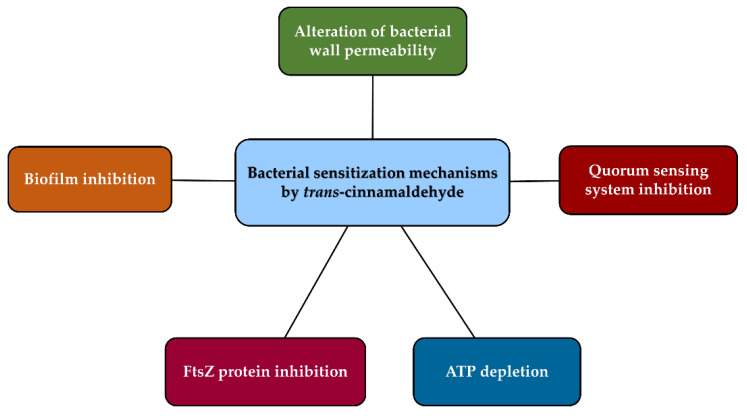
Possible mechanisms underlying the bacterial sensitizing properties of *trans*-cinnamaldehyde.

In regard to the mechanisms accounting for the bacterial sensitizing properties of cinnamaldehyde, the substance has been shown to affect multiple targets, including the bacterial wall, biofilm, quorum sensing system, cell metabolism, and factors involved in cell survival (Figure 4), which in turn can contribute to the potentiation of the antibiotic efficacy and the overcoming of resistance. 

As also reported for other essential oil compounds [21,22], Shi et al. [59] hypothesized that the synergistic effects of cinnamaldehyde in combination with nisin in *S. aureus* ATCC 29213 could arise from its ability to damage the bacterial wall and alter its permeability, thus affecting the antibiotic absorption and impairing the bacterial cell homeostasis, leading to autolysis and cell death. Indeed, an 54.5% membrane damage was induced by the combination of cinnamaldehyde and nisin with respect to the drug alone (28% damage) [59]. Similarly, Chadha et al. [65] hypothesized that cinnamaldehyde can cause membrane permeabilization and disruption along with oxidative damage, thus facilitating the penetration of the antibiotic gentamicin into cell and making the bacterial cell more susceptible to its antimicrobial activity. 

Wang et al. [61] highlighted that the substance was able to destroy the bacterial wall and biofilm of MRSA and to downregulate the transcription and translation of the antibiotic resistance gene mecA; these effects could explain the synergism with non-beta-lactam antibiotics. Dhara et al. [62] also reported alterations in the cell surface morphology, shrinkage of the cell surface, and cytoplasm lowering in Gram-negative bacteria, i.e., *E. coli* and *K. pneumoniae*, after treatment with cinnamaldehyde, likely as a consequence of permeability and osmotic changes induced by the substance. Moreover, deep pores, disruption of the cytoplasmic membrane, and decomposition of inner organelles on cell surfaces were revealed after treatment with the combination of cinnamaldehyde and cefotaxime/ciprofloxacin [62]. 

Gram-negative bacteria carry an outer membrane characterized by an asymmetric hydrophobic bilayer composed of phospholipids and lipopolysaccharides (LPS), the latter playing a crucial role in the bacteria’s protection [83]. Most antibiotics are absorbed through the outer membrane to reach their targets: hydrophobic drugs are able to pass the membrane by diffusion mechanisms, while hydrophilic ones, like β-lactams, exploit the bacterial porins to be transferred into cells [84]. Any alteration in the outer membrane of Gram-negative bacteria, including changes in the hydrophobic properties and porin mutations, can lower the antibiotic permeability, thus leading to bacterial resistance [84]. In this respect, the results obtained by Dhara et al. [62] strengthen the hypothesis that an impairment in the bacterial wall by cinnamaldehyde is a key mechanism of its bacterial sensitizing activity.

Some studies also highlighted that cinnamaldehyde significantly inhibited the biofilm formation and the expression of the biofilm regulatory gene *hld* in methicillin-resistant *Staphylococcus aureus* [61]. Moreover, the combined treatments of cinnamaldehyde with colistin and tobramycin potentiated the drug’s ability to inhibit biofilm formation, leading to a complete inhibition of the process [64]. 

Biofilm is composed by a complex community of microbes that can adhere to a surface or form aggregates, enclosed in an extracellular polysaccharide matrix [85,86]. It enhances the bacterial resistance to hostile environmental conditions, allows the cellular exchange of plasmids encoding for antibiotic resistance, and impairs the activation of the immune system response, thus favoring the bacterial invasion [86]. It is also responsible for the development of persistent infections [87]. The biofilm inhibition by cinnamaldehyde can arise from different mechanisms, among which is a block of the quorum sensing system (QS), as recently highlighted by Chadha et al. [65] in combination with gentamicin in *P. aeruginosa*.

QS represents a cell-to-cell communication mechanism that occurs extensively in both Gram-positive and Gram-negative bacteria [88]. It consists of enzymes, receptors, and factors that regulate various bacterial functions, including biofilm production, sporulation, motility, and virulence [88,89]. The QS signal molecules are characterized by a low molecular weight and can be classified into different classes, including acyl homoserine lactones (AHLs), furanosyl borate diesters (AI2), cis-unsaturated fatty acids (DSF family signals), and peptides [89]. In *P. aeruginosa*, the QS system harbors two complete AHL circuits, namely LasI/LasR and RhlI/RhlR, with LasI/R being hierarchically positioned upstream of the RhlI/R circuit [88].

Chadha et al. [65] reported that cinnamaldehyde was able to affect the QS system in *P. aeruginosa* PAO1 by downregulating the QS and virulence genes (e.g., las, rhl, rhlAB, aprA, toxA, plcH) and abrogating the biosynthesis of AHL (acyl-homoserine lactones) molecules, involved in the QS processes. Similarly, Topa et al. [74] showed that cinnamaldehyde inhibited the expression of the LasB, RhlA, and PqsA QS systems in *P. aeruginosa*. Cinnamaldehyde exhibited a quorum quenching (QQ) potential, being able to affect the QS system at subinhibitory concentrations [65]. As also confirmed by molecular docking studies, the effect can be attributed to the ability of the substance to easily gain access to the active site of the QS receptors of *P. aeruginosa*, owing to its relatively small size; furthermore, being structurally similar to the AHL molecules, 3-oxo-C12-HSL and C4-HSL, it can strongly interact with the QS receptors, thus attenuating the QS circuits, inhibiting the biofilm formation, and lowering the bacterial virulence and motility [65]. Particularly, it has been hypothesized that cinnamaldehyde may abrogate the twitching motility in *P. aeruginosa* by inhibiting the mechanotactic functions of type IV pilus and the swimming and swarming motilities because of its anti-QS properties [65]. Furthermore, an inhibition of the EPS (extracellular polymeric substance) production by cinnamaldehyde, especially in relation to the alginate and rhamnolipid components, seems to directly modulate the pseudomonal biofilm formation and demonstrates the anti-fouling properties of the natural substance against *P. aeruginosa* [65].

Other mechanisms have also been proposed to explain the synergistic effects of cinnamaldehyde in combination with antibiotics. Particularly, Thirapanmethee et al. [90] showed that the substance blocked the polymerization, assembly, and bundling of the bacterial protein FtsZ in *Acinetobacter baumanni*, involved in the control of cell division [90,91]. Furthermore, some studies highlighted an ATP depletion by cinnamaldehyde [92,93], which could be reflected in an impairment of the bacterial function and survival. 

## 5. Conclusions

Altogether, the collected evidence suggests a possible interest in cinnamaldehyde as an adjuvant strategy to synergize or support the effects of synthetic antibiotics against bacteria, especially against resistant strains and superbugs. However, as a small and heterogeneous group of in vitro studies, more in-depth mechanistic evidence and clinical investigations should be encouraged to clarify the promises and challenges of cinnamaldehyde in antibiotic resistance.

## Figures and Tables

**Table 1:** Natural occurrence of *trans*-cinnamaldehyde in plant essential oils.

Plant Species/Family	Plant Part	*trans*-Cinnamaldehyde (%)	Ref.
*Chrysanthemum viscidehirtum* Schott Tell/Lauraceae	Leaf	2.1	[30]
	Aerial parts	0.7	
*Cinnamomum angustifolium* Lukman/Lauraceae	Leaf and bark	0.2	[28]
*Cinnamomum aureofulvum* Gamble/Lauraceae	Bark	46.6	[31,32]
*Cinnamomum burmannii* Nees & T. Nees/Lauraceae	Leaf	45–62	[33]
	Bark	17–32	
*Cinnamomum cassia* Nees/Lauraceae	Bark	85	[28,34,35]
*Cinnamomum curvifolium* Nees/Lauraceae	Leaf	8.9	[36]
	Steam bark	1.2	
*Cinnamomum durifolium* Kosterm/Lamiaceae	Aerial parts	0.6	[37]
*Cinnamomum loureirii* Nees/Lauraceae	Bark	50.2–92.9	[38,39]
*Cinnamomum mairei* H. Léveillé/Lauraceae	Leaf	1.9	[36]
	Steam bark	6.5	
*Cinnamomum osmophloeum* Kaneh/Lauraceae	Leaf	79.8	[40,41]
*Cinnamomum pubescens* Kochummen/Lauraceae	Leaf	56.1	[42]
*Cinnamomum sericans* Hance/Lauraceae	Leaf	0.6	[37]
*Cinnamomum tamala* Nees Eberm/Lauraceae	Leaf Bark	68.7–79.4 64.8	[43,44] [43]
*Cinnamomum verum* J. Presl/Lauraceae	Leaf	0.6	[28,45]
	Bark	89.3	
*Cinnamomum zeylanicum* Blume/Lauraceae	Bark	44.2–68.7	[32,35,46,47]
	Leaf	1–5	[46]
*Marrubium astracanicum* Jacq./Lauraceae	Aerial parts	2.2	[48]
*Psidium cattleianum* Sabine/Lamiaceae	Aerial parts	2.2	[49]
	Fruit	0.6	
*Teucrium persicum* Boiss/Myrtaceae	Aerial parts	0.4	[48]

## Data Availability

Not applicable.
